# Adenoviral Gene Transfer of PLD1-D4 Enhances Insulin Sensitivity in Mice by Disrupting Phospholipase D1 Interaction with PED/PEA-15

**DOI:** 10.1371/journal.pone.0060555

**Published:** 2013-04-09

**Authors:** Angela Cassese, Gregory A. Raciti, Francesca Fiory, Cecilia Nigro, Luca Ulianich, Ilenia Castanò, Vittoria D’Esposito, Daniela Terracciano, Lucio Pastore, Pietro Formisano, Francesco Beguinot, Claudia Miele

**Affiliations:** 1 Dipartimento di Scienze Mediche e Traslazionali, Università di Napoli “Federico II” and Istituto di Endocrinologia e Oncologia Sperimentale Gaetano Salvatore, Consiglio Nazionale delle Ricerche, Naples, Italy; 2 Dipartimento di Biochimica e Biotecnologie Mediche, Università di Napoli “Federico II”, Naples, Italy; 3 CEINGE-Biotecnologie Avanzate, Naples, Italy; University of Illinois at Chicago, United States of America

## Abstract

Over-expression of phosphoprotein enriched in diabetes/phosphoprotein enriched in astrocytes (PED/PEA-15) causes insulin resistance by interacting with the D4 domain of phospholipase D1 (PLD1). Indeed, the disruption of this association restores insulin sensitivity in cultured cells over-expressing PED/PEA-15. Whether the displacement of PLD1 from PED/PEA-15 improves insulin sensitivity *in vivo* has not been explored yet. In this work we show that treatment with a recombinant adenoviral vector containing the human D4 cDNA (Ad-D4) restores normal glucose homeostasis in transgenic mice overexpressing PED/PEA-15 (Tg _ped/pea-15_) by improving both insulin sensitivity and secretion. In skeletal muscle of these mice, D4 over-expression inhibited PED/PEA-15-PLD1 interaction, decreased Protein Kinase C alpha activation and restored insulin induced Protein Kinase C zeta activation, leading to amelioration of insulin-dependent glucose uptake. Interestingly, Ad-D4 administration improved insulin sensitivity also in high-fat diet treated obese C57Bl/6 mice. We conclude that PED/PEA-15-PLD1 interaction may represent a novel target for interventions aiming at improving glucose tolerance.

## Introduction

Type 2 Diabetes (T2D) is a chronic disorder associated with vascular complications and increased risk of cardiovascular disease [1]. Largely because of its contribution to cardio-vascular disease, T2D is also associated to excess mortality. The prevalence of T2D is increasing, making the identification of innovative therapeutic targets an important priority in this field [2], [3].

We recently described that overexpression of the *PED/PEA-15* gene is a common abnormality in T2D. *PED/PEA-15* mRNA and protein levels are highly expressed in skeletal muscle and adipose tissues from individuals with T2D and their First Degree Relatives (FDR) compared with euglycaemic individuals [4], [5]. In addition, high PED/PEA-15 levels strongly correlate with resistance to insulin action in the lean mass of these individuals [5]. Transgenic mice featuring ubiquitous over-expression of *Ped/pea-15* (Tg_Ped/pea-15_) exhibit decreased glucose tolerance, due to a combination of fat and skeletal muscle insulin resistance and reduced insulin secretion in response to glucose. These mice develop diabetes when treated with a high fat diet (HFD) [6]. In previous studies, we have shown that PED/PEA-15 over-expression impairs both *in vitro* and *in vivo* insulin-stimulated glucose disposal through the interaction with the D4 domain of the Phospholipase D1 (PLD1). Both PLD1 stability and protein expression are increased in cells overexpressing PED/PEA-15, as well as the PLD1-dependent activation of the protein kinase Calpha (PKCalpha) [6]–[8]. This, in turn, causes inhibition of the insulin-mediated PKCzeta activity, and decreased insulin-induced GLUT4 translocation to the plasma membrane [6]–[8]. In addition, we showed that in cultured muscle cells stably overexpressing PED/PEA-15, the expression of D4 displaces the interaction of PED/PEA-15 with PLD1. This effect reduces basal PKCalpha activity and restores the insulin-stimulated glucose disposal mediated by PKCzeta [9].

In the present work, we have used a recombinant adenoviral vector containing the human D4 cDNA (Ad-D4) to establish whether the dissociation of PLD1 from PED/PEA-15 improves whole body glucose tolerance, in both Tg_Ped/pea-15_ and obese high fat-fed C57BL6 mice.

## Materials and Methods

### Ad-D4 Cloning and Preparation

The Ad-D4 vector was obtained as previously described by [10]. Briefly, human D4 cDNA was introduced at the HindIII and KpnI sites in pAd-Track-CMV shuttle vector, which contains two distinct gene cassettes for the simultaneous expression of the target protein and of the Green Fluorescent Protein (GFP) (kindly provided by Dr. Bert Vogelstein). The resulting pAd-Track-CMV-D4 plasmid was then linearized by PmeI and transformed with the adenoviral pAdEasy-E1 vector in BJ5183 EC cells. The resulting recombinant vector was linearized with PacI and transfected in HEK293 cells using lipofectamine reagent (Invitrogen). 24 h after transfection, cells were overlaid with agarose to permit isolation of individual virus plaques, and visible plaques were selected and expanded after 7–10 days from transfection. Viruses were then purified from HEK293 cells by freeze/thaw cycles and cesium chloride step gradients, and aliquots of the obtained recombinant adenoviruses were frozen and stored at −80°C.

### Cell Culture Procedures, Adenoviral Infection and 2-[1-3H]deoxy-d-glucose Uptake *in vitro*

Parental L6 rat skeletal muscle cells (L6Wt) and L6 cells over-expressing PED/PEA-15 (L6PED/PEA-15) were grown in DMEM supplemented with 10% (vol./vol.) FBS, 2 mmol/l L-glutamine and antibiotics. L6 myotubes were allowed to differentiate as described previously [11]. Differentiated L6 myotubes were then infected with Ad-D4 or Ad-GFP vector by infections with different concentrations of the virus. Subsequent experiments were performed 48 h after initial addition of virus. Cell lysates and immunoblotting were carried out as described previously [11]. 2-DG uptake was measured as reported previously [11].

#### Mouse models

Tg_Ped/pea-15 _mice were backcrossed for 8 generations on the C57/Bl6 background. 3–6 months old male and female Tg_Ped/pea-15 _mice and their normal littermates (wild type; WT) were used for all studies. Mice were housed in the animal facilities for at least one week before experiments. All procedures and euthanasia were conducted in accordance with adoption of Institutional Animal Care and Utilization Committee (Ministero della Salute, Dipartimento della Sanità Pubblica Veterinaria, della Sicurezza Alimentare e degli Organi Collegiali per la Tutela della Salute, Direzione Generale della Sanità Animale e dei Farmaci Veterinari). The ethics committee of the Ministero della Salute, Dipartimento della Sanità Pubblica Veterinaria, della Sicurezza Alimentare e degli Organi Collegiali per la Tutela della Salute Direzione Generale della Sanità Animale e dei Farmaci Veterinari approved this study. Mice were housed one per cage on a 12-h light/dark cycle (lights on 0600–1800) and fed water and standard diet (11% calories from fat; Research Diets formulas D12328; Research Diets, Inc., New Brunswick, N.J) *ad libitum* (AL). For the high fat diet (HFD), 2-month-old WT (n = 12) mice were fed with a 60 kcal% fat content (Research Diets formulas D12331; Research Diets, Inc., New Brunswick, N.J.) ad libitum for 11 weeks.

#### Ad-D4 infection *in vivo*

The day of adenoviral injection, mice were anaesthetized by i.p. administration of 2,2,2-tribromoethanol (Sigma Aldrich), and then the Ad-D4 or the control Ad-GFP vectors were i.v. injected into the tail vein at the final concentration of 2×10^12^ vp/kg. Mice were euthanized after 6 days from infection, and their tissues were quickly collected and frozen in dry-ice.

#### Lysate preparation, Immunoprecipitation (IP) and Western Blot (WB)

Mice were fasted overnight, anesthetized, and injected i.p. with saline or insulin (10 U/kg body weight). Ten min after injection, skeletal muscle tissues (quadriceps, gastrocnemious and tibialis) were removed and frozen in dry ice. Tissues homogenates and cell lysates were separated by SDS-PAGE and analyzed by western blot as previously described [12]. For each IP experiment about 0.5–1 mg of skeletal muscle protein lysate was precleared with 20 µl of pre-immune serum at 4°C for 30 min and then incubated with 1.5 µg of anti-PED-PEA15 or anti-PLD1 antibody O.N. at 4°C. After incubation, 40 µl of protein A sepharose (Invitrogen) in a 1∶1 mixture of protein A sepharose and Lysis Buffer were added to the IP samples. Purified interacting complex was released from protein A sepharose beads by boiling in SDS-PAGE sample buffer and resolved on SDS-PAGE. Samples were then subjected to WB analysis. Membranes were firstly probed with antibodies to PED/PEA-15 antiserum, PLD1 (Cell Signalling Technology), phospho-PKCalpha, PKCalpha (Millipore), phospho-PKCzeta, PKCzeta, Tubulin (Santa Cruz Biotech Inc), and GFP-peptide living colors (BD Transduction Laboratories), and then with secondary mouse or rabbit antibodies (GE Healthcare) before detection of the signal with ECL plus (GE Healthcare).

#### Real time quantitative PCR

Total RNA extraction, cDNA synthesis and Real Time quantitative PCR were performed as described [13]. Primer sequences are as follows: D4 F: 5′-agtccatccacgccgttac-3′; D4 R: 5′-tcctctgggcaatggcatcg-3′; GAPDH F: 5′-gccttccgtgttcctacc-3′; GAPDH R: 5′-agagtgggagttgctgttg-3′.

#### PLD assay *in vitro*

PLD activity assays were carried out using the *in vitro* head group release assay and the *in vivo* transphosphatidylation assay as described previously [8]. Briefly, skeletal muscle homogenates were centrifuged at 10,000×g for 5 min, and their supernatants were designated as crude membranes. PLD1 activity was then determined by measuring the transphosphatidylation activity in the presence of butanol. Recombinant ADP-ribosylation factor 1 (ARF1) and Ras homolog gene family, member A (RhoA) were added to the samples and were activated using 10 µM of Guanosine 5′-[γ-thio]-triphosphate (GTP[S]). Lipid products were extracted and then separated on a Silica Gel 60 TLC plate with chloroform/methanol/acetic acid (13∶3∶1 by vol.) as the developing solvent. The spots corresponding to [^14^C] phosphatidyl-butanol were determined by autoradiography and their radioactivity was counted to obtain analytical values.

#### Metabolite assays

Blood glucose levels were measured with glucometers (OneTouch Ultra2, LifeScan); insulin was measured by radioimmunoassay with rat insulin as standard (Linco Research). Fasting plasma free fatty acids were measured with the Wako NEFA C kit (Wako Chemicals), and tricylglycerol was measured with the Infinity triglyceride reagent (Sigma Aldrich).

#### Glucose and insulin tolerance, and measurement of 2-[1-3H] deoxy-D-glucose uptake

Glucose tolerance tests (GTTs) and insulin tolerance tests (ITTs) were measured as described [13]. For analyzing glucose utilization, an intravenous injection of 1 µCi of the nonmetabolizable glucose analog 2-[1-3H]deoxy-d-glucose (2-DG) (Amersham Pharmacia Biotech) and an intraperitoneal injection of insulin (0.75 mU/g body wt) were administered to random fed mice. The specific blood 2-DG clearance was determined with 25-µl blood samples (tail vein) obtained 1, 15, and 30 min after injection as previously reported [13]. Quadriceps, soleus and tibialis skeletal muscles and perigonadal adipose tissue were removed 30 min after the injections. Glucose utilization index was determined by measuring the accumulation of radiolabeled compound [13]. The amount of 2-DG-6 phosphate per milligram of protein was then divided by the integral of the concentration ratio of 2-DG to the measured unlabeled glucose. Glucose utilization indexes were expressed as picomoles per milligram of protein per minute.

#### Statistical analysis

Data are expressed as means ± SEM and statistical significance between groups was analyzed by 2-tailed Student’s t-test or analysis of variance (ANOVA) as appropriate. *P* values of <0.05 were considered statistically significant. The total AUC and the inverse AUC for glucose response during GTT and ITT were calculated as [12].

## Results

### 

#### Ad-D4 Transduction *in vitro* and *in vivo*

**Figure 1:**
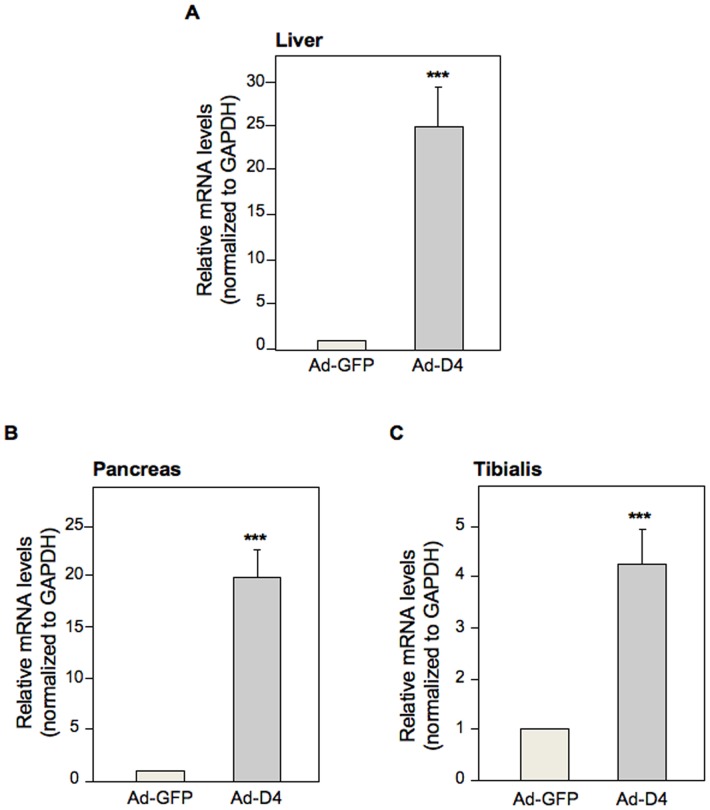
D4 mRNA expression in Tg_Ped/pea-15 _mice. *D4* mRNA expression was determined by quantitative Real Time RT-PCR analysis of total RNA isolated from liver (**A**), pancreas (**B**), and tibialis skeletal muscle tissue (**C**) of Tg_Ped/pea-15 _mice at one week post Ad-D4 or Ad-GFP infection. GAPDH was used as housekeeping gene. mRNA levels in Ad-D4 treated Tg_Ped/pea-15 _mice are relative expression units to those in Ad-GFP treated Tg_Ped/pea-15 _mice used as control (mean ± SEM; n = 3). ***p<0.001 vs Ad-GFP treated Tg_Ped/pea-15_.

Untrasfected L6 skeletal muscle cells (L6_Wt_) and L6 cells over-expressing PED/PEA-15 (L6_PED/PEA-15_) were transduced with 4 different clones of Ad-D4 vector. D4 levels were indirectly evaluated by measuring the Green Fluorescent Protein (GFP) expression, being the GFP expressed by the Ad-D4 vector. At 48 hours post-transduction, GFP production was detectable in both L6_Wt_ and L6_PED/PEA-15_ cells transfected with clone 1; lower levels were detectable when the cells were infected with clones 2, 3 and 4 (Figure S1a and b). Then, the biological activity of Ad-D4 vector was analyzed by measuring insulin-induced glucose uptake in the insulin resistant L6_PED/PEA-15_ cells transduced with clone 1 or with the adenoviral vector expressing only the GFP (Ad-GFP) as a control. Transduction of L6_PED/PEA-15_ cells with Ad-D4 induced a 2-fold increase of the 2-DG uptake compared to L6_PED/PEA-15_ cells which were not trasduced (NT) or to L6_PED/PEA-15_ transduced with Ad-GFP (Figure S1c), indicating that Ad-D4 transduction restores insulin sensitivity in L6_PED/PEA-15_ cells. D4 was then transduced *in vivo* by injecting Tg_Ped/pea-15 _mice with Ad-D4 or Ad-GFP vector. D4 mRNA expression was measured in different tissues upon 6 days after the adenoviral delivery. D4 mRNA resulted highly expressed in liver, pancreas as well as in tibialis skeletal muscle of the Ad-D4 injected transgenic mice compared to the Ad-GFP injected control mice (Figure 1a, b, c).

#### Ad-D4 treatment improves glucose homeostasis and metabolic parameters in Tg_Ped/pea-15 _mice

**Figure 2:**
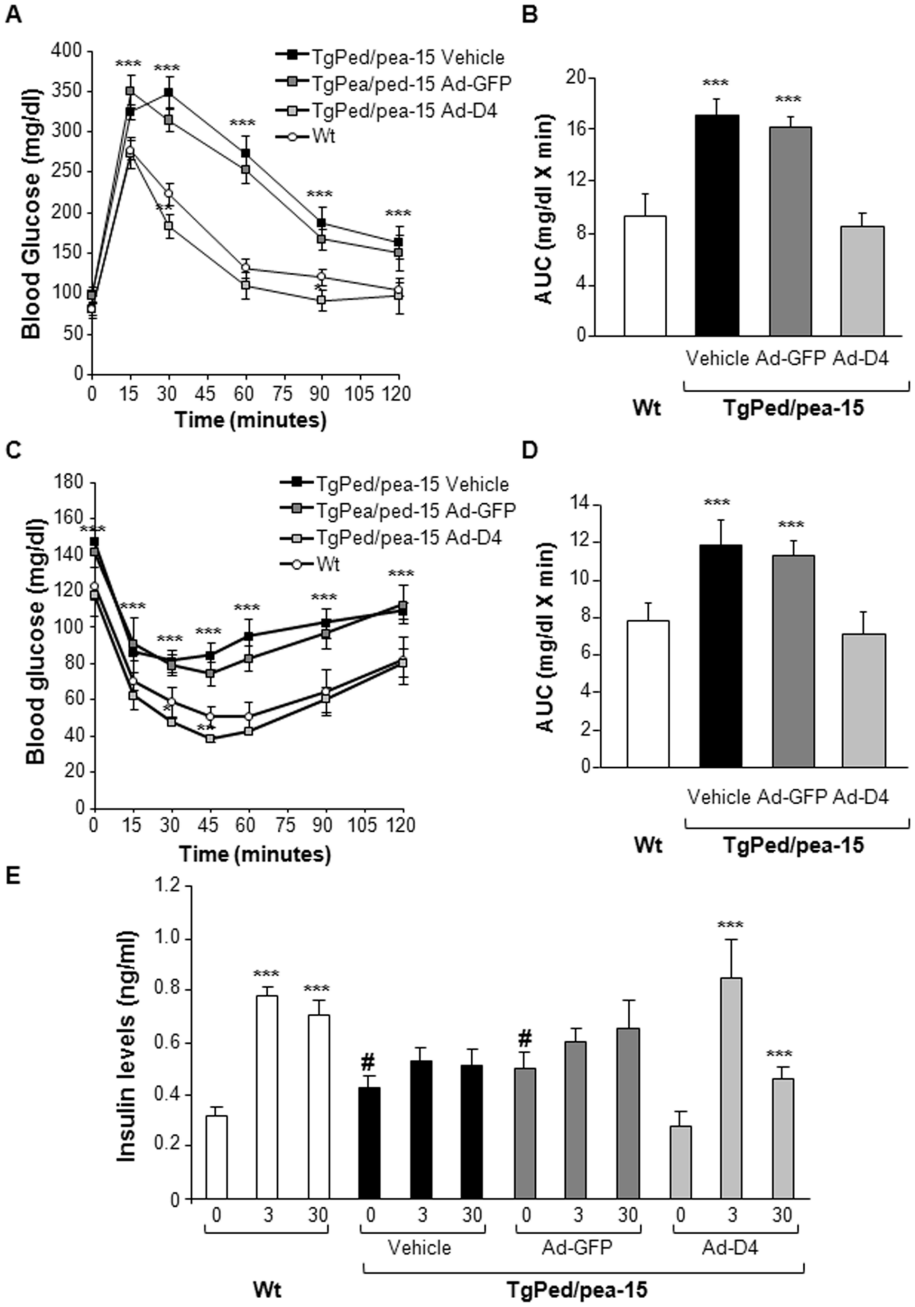
Glucose tolerance, insulin sensitivity and insulin secretion in Tg_Ped/pea-15 _mice. **A)** Glucose Tolerance Test (GTT) and (**B**) Area Under the Curve (AUC) glucose in Tg_Ped/pea-15 _mice treated with vehicle (black square and column) or after infection with Ad-GFP (dark grey square and column) or with Ad-D4 (light grey square and column), and wild type mice (Wt, white circle and column) used as controls. **C)** Insulin Tolerance Test (ITT) and (**D**) Area Under the Curve (AUC) glucose in Tg_Ped/pea-15 _mice treated with vehicle (black square and column) or after infection with Ad-GFP (dark grey square and column) or with Ad-D4 (light grey square and column), and wild type mice (Wt, white circle and column) used as controls. For each experiment, values are expressed as means ± SEM of determinations in at least eight mice per group. ***p<0.001 vs Wt. **E)** Glucose-induced insulin secretion in 6 Tg_Ped/pea-15 _mice treated with vehicle (black square and column) or after infection with Ad-GFP (dark grey square and column) or with Ad-D4 (light grey square and column), and wild type mice (Wt, white circle and column). Data points represent the means ± SEM of determinations in five mice per group. ***p<0.001, t = 3 and t = 30 vs t = 0; #p<0.001, Tg_Ped/Pea-15 _t = 0 vs Wt t = 0.

We then aimed at evaluating the effect of D4 on glucose tolerance. As previously reported [6], Tg_Ped/pea-15 _mice exhibited increased fasting and random fed blood glucose levels and fasting insulin and NEFA concentrations (Table 1). Furthermore, glucose loading (2 g/kg body weight) made these mice significantly more hyperglycaemic than control mice during the following 120 min (Fig. 2a, b). When injected with the Ad-D4 vector, Tg_Ped/pea-15**_mice showed fasting and random fed blood glucose levels comparable to those of control mice (Table 1), and exhibited normal glucose tolerance after GTT (Figure 2a, b). The Ad-D4 treatment was also able to lower fasting insulin and NEFA concentrations in Tg_Ped/pea-15 _mice, achieving values similar to those of control mice (Table 1). No improvement of either fasting and random fed blood glucose levels, fasting insulin and NEFA levels, and glucose tolerance were observed when Tg_Ped/pea-15 _mice were treated with the Ad-GFP (Table 1; Figure 2a, b). Tg_Ped/pea-15 _mice injected with both the Ad-D4 and Ad-GFP showed a slight increase in fasting triacyglycerol levels when compared to both the control and Tg_Ped/pea-15 _mice before adenoviral delivery (Day 0; Table 1), suggesting that this effect was not due to D4 transduction.

**Table 1:** Metabolic characteristics of TgPed/pea-15 mice.

Metabolic parameters	Wild Type	TgPed/pea-15		
	Vehicle	Vehicle	Ad-GFP	Ad-D4
*Fasting glucose (mg/dl)*	81.8±7.0	99.6±3.6^a^	97.3±9.7^b^	79.8±7.6e,g
*Fed glucose (mg/dl)*	126.3±2.0	140.9±6.5^a^	148.9±8.6^b^	117.5±16.2f,h
*Fasting insulin (ng/ml)*	0.32±0.03	0.45±0.05^a^	0.50±0.06^b^	0.28±0.06f,h
*Fasting NEFA (mEq/l)*	0.62±0.12	0.81±0.05^a^	0.85±0.08^b^	0.56±0.04f,h
*Fasting Triacylglycerol (mg/ml)*	0.23±0.03	0.27±0.02	0.36±0.03b,c	0.38±0.03d,f

Data are means ± SEM of determinations in at least 10 mice per group. Vehicle TgPed/pea-15 mice vs. Vehicle Wild Type mice;

a *P*<0.001. Ad-GFP TgPed/pea-15 mice vs. Vehicle Wild Type mice;

b *P*<0.001. Ad-GFP TgPed/pea-15 mice vs. Vehicle TgPed/pea-15 mice;

c *P*<0.001. Ad-D4 TgPed/pea-15 mice vs. Vehicle Wild Type mice;

d *P*<0.001. Ad-D4 TgPed/pea-15 mice vs. Vehicle TgPed/pea-15 mice;

e *P*<0.01 and

f *P*<0.001. Ad-D4 TgPed/pea-15 mice vs. Ad-GFP TgPed/pea-15 mice;

g *P*<0.01 and

h *P*<0.001.

#### Ad-D4 treatment improves insulin sensitivity and secretion in Tg_Ped/pea-15 _mice

To verify whether the beneficial effect of Ad-D4 treatment on glucose tolerance is also accompanied by an improvement of whole body insulin sensitivity, we performed insulin tolerance test (ITT). Following i.p. injection of insulin (0.75 U/kg body weight) a strong reduction of the hypoglycaemic response was observed in untreated Tg_Ped/pea-15 _mice compared to controls (Fig. 2c, d). On the other hand, insulin was able to lower glucose levels during ITT in the Ad-D4 treated Tg_Ped/pea-15 _mice to levels comparable to control mice, but not in the Ad-GFP treated Tg_Ped/pea-15 _mice (Fig. 2c, d). We then evaluated glucose-induced insulin secretion. In control mice, a 2.5 fold increase in insulin secretion was observed 3 min after glucose injection, with levels remaining higher than baseline for up to 30 min, indicating a second-phase response (Fig. 2e). In contrast, the acute first-phase insulin secretory response to glucose and the late second-phase response were completely abolished in Tg_Ped/pea-15 _mice (Fig. 2e). Interestingly, Ad-D4 treatment improved the insulin secretion in response to glucose loading in Tg_Ped/pea-15 _mice, while Ad-GFP treatment did not (Fig. 2e). Thus, Ad-D4 administration ameliorated both insulin sensitivity and secretion in Tg_Ped/pea-15 _mice, restoring a normal glucose homeostasis.

#### Ad-D4 treatment re-establishes insulin sensitivity in skeletal muscle tissue of Tg_Ped/pea-15 _mice

**Figure 3:**
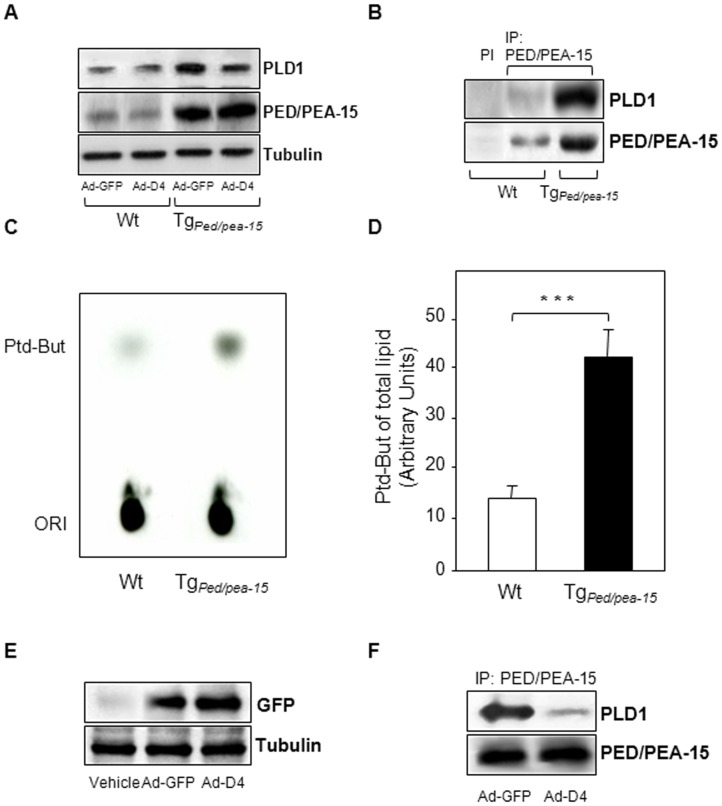
Protein interaction of PED/PEA-15 with PLD1 and PLD1 activity in Tg_Ped/pea-15 _mice. **A)** Immunoblots of total protein lysates from skeletal muscle homogenates of wild type (Wt) and Tg_Ped/pea-15 _mice at one week post Ad-D4 or Ad-GFP infection. The blots were probed with anti-PLD1, anti-PED/PEA-15 and anti-Tubulin antibodies. PI stands for pre-immune serum. **B)** Immunoblots of immunoprecipitated from skeletal muscle homogenates of wild type (Wt) and Tg_Ped/pea-15 _mice. IP were performed using the anti-PED/PEA-15 antibody as described in *Experimental Procedures*. The upper blot was probed with anti-PLD1 antibody, while the bottom blot was striped and then probed with anti-PED/PEA-15 antibody. **C)** PLD1 activity was analyzed in skeletal muscle homogenates from wild type (Wt) and Tg_Ped/pea-15 _mice by measuring the transphosphatidyl-butanol levels as described in *Materials and methods.* The autoradiography shown is representative of three independent assays. **D)** The bar graph represents the densitometric quantization of the spots in three experiments in triplicate. ***p<0.001 vs. Wt. **E)** Immunoblots of whole lysates from skeletal muscle homogenates of Tg_Ped/pea-15 _mice at one week post Ad-D4 or Ad-GFP infection or PBS injection (vehicle). The upper blot was probed with anti-GFP antibody, while the bottom blot was striped and then probed with anti-tubulin antibody. **F)** Immunoblots of immunoprecipitated from skeletal muscle homogenates of Tg_Ped/pea-15 _mice at one week post Ad-D4 or Ad-GFP infection. IP were performed using the anti-PED/PEA-15 antibody. The upper blot was probed with anti-PLD1 antibody, while the bottom blot was striped and then probed with anti-PED/PEA-15 antibody.

**Figure 4:**
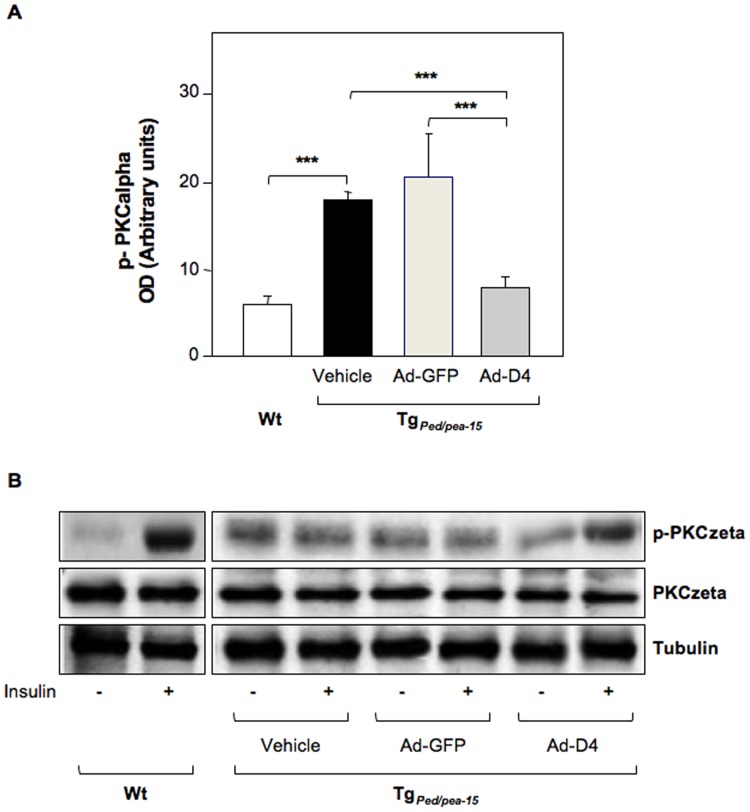
Effect of D4 on PKCalpha and zeta activation in Tg_Ped/pea-15 _mice. PKCalpha (**A**) and zeta (**B**) activations were determined in the skeletal muscle tissues from Tg_Ped/pea-15 _mice at one week post Ad-D4 or Ad-GFP infection. Tg_Ped/pea-15_ or wild type (Wt) mice injected with PBS (vehicle) were used as control. For the experiment, mice were fasted over night and then i.p. injected or not with insulin (10 U/kg body weight) 10 min before determination. **A)** The bar graph represents the densitometric quantization of phospho-PKCalpha in three independent immunoblots. ***p<0.001 vs. Wt. **B)** The corresponding blots show the levels of PKCzeta (total and phosphorilated forms) and tubulin in mice as indicated. Blots are representative of three independent experiments.

**Figure 5:**
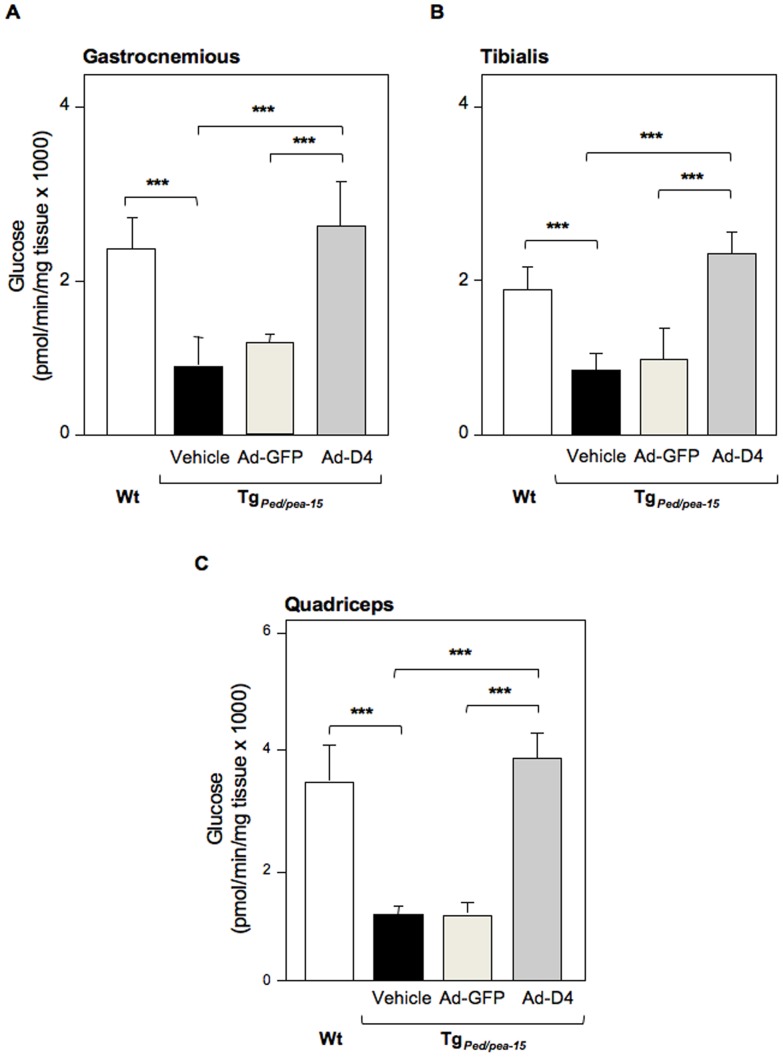
Glucose uptake in skeletal muscle tissues of Tg_Ped/pea-15 _mice. Ex vivo Glucose uptake into gastrocnemius (**A**), tibialis (**B**) and quadriceps (**C**) skeletal muscle of Tg_Ped/pea-15 _mice treated with vehicle (black column) or after infection with Ad-GFP (light grey column) or with Ad-D4 (dark grey column), and wild type mice (Wt, white column) used as controls. Values are expressed as means ± SEM of determinations in at least five mice per group. ***p<0.001 vs Wt.

We then evaluated the expression of both PED/PEA-15 and PLD1 and whether PED/PEA-15/PLD1 interaction also occurs *in vivo*. To this aim, we performed western blot analysis to evaluate the total protein amount and co-immunoprecipitation experiments in tibialis skeletal muscle extracts from Tg_Ped/pea-15_ and control mice (Wt). As expected, we found that both PED/PEA-15 and PLD1 protein levels were increased in tibialis skeletal muscle extracts from Ad-GFP treated Tg_Ped/pea-15_ compared to control mice (Wt). PLD1 levels were reduced by D4 expression in Tg_Ped/pea-15_ mice, wilst D4 did not alter PED/PEA-15 expression (Figure 3a). PED/PEA-15 interacted with PLD1 in the tibialis muscles of both Wt and transgenic mice, and this interaction was increased by 4-fold in Tg_Ped/pea-15 _mice (Figure 3b). PLD1 activity was also significantly increased in muscle homogenates from Tg_Ped/pea-15 _mice (Figure 3c, d). Following Ad-D4 infection, D4 expression was confirmed by the detection of a 28 KDa protein corresponding to GFP in the tibialis muscle extracts from both Ad-GFP and Ad-D4 infected transgenic mice (Figure 3e). The amount of PLD1 protein co-immunoprecipitated with PED/PEA-15 protein was significantly reduced in the tibialis muscle homogenate from the Tg_Ped/pea-15 _mice injected with Ad-D4 (Figure 3f). We further investigated whether the ability of D4 to prevent PED/PEA-15/PLD1 interaction in these samples restores PKCalpha and zeta activation. Basal PKCalpha phosphorylation was increased in the tibialis muscle from Tg_Ped/pea-15**_mice injected with either vehicle or Ad-GFP when compared to that of Wt mice, while Ad-D4 treatment lowered PKCalpha activity to levels similar to those of control mice (Figure 4a). In addition, whilst insulin failed in inducing PKCzeta phosphorylation in the muscle of Tg_Ped/pea-15 _mice injected with either vehicle or Ad-GFP, insulin-induced PKCzeta activation following Ad-D4 infection was restored to levels comparable to those of control mice (Figure 4b). Finally, insulin-stimulated glucose transport was strongly reduced in the gastrocnemious as well as in the tibialis and quadriceps skeletal muscle of Tg_Ped/pea-15**_mice injected with either vehicle or Ad-GFP compared to control mice, whilst infection of Ad-D4 resulted in a marked increase of the glucose uptake to levels comparable to those of controls (Figure 5a, b, c). Thus, Ad-D4 delivery fully restored insulin sensitivity in Tg_Ped/pea-15 _mice.

#### Ad-D4 treatment improves insulin sensitivity in obese C57BL/6 mice

**Figure 6:**
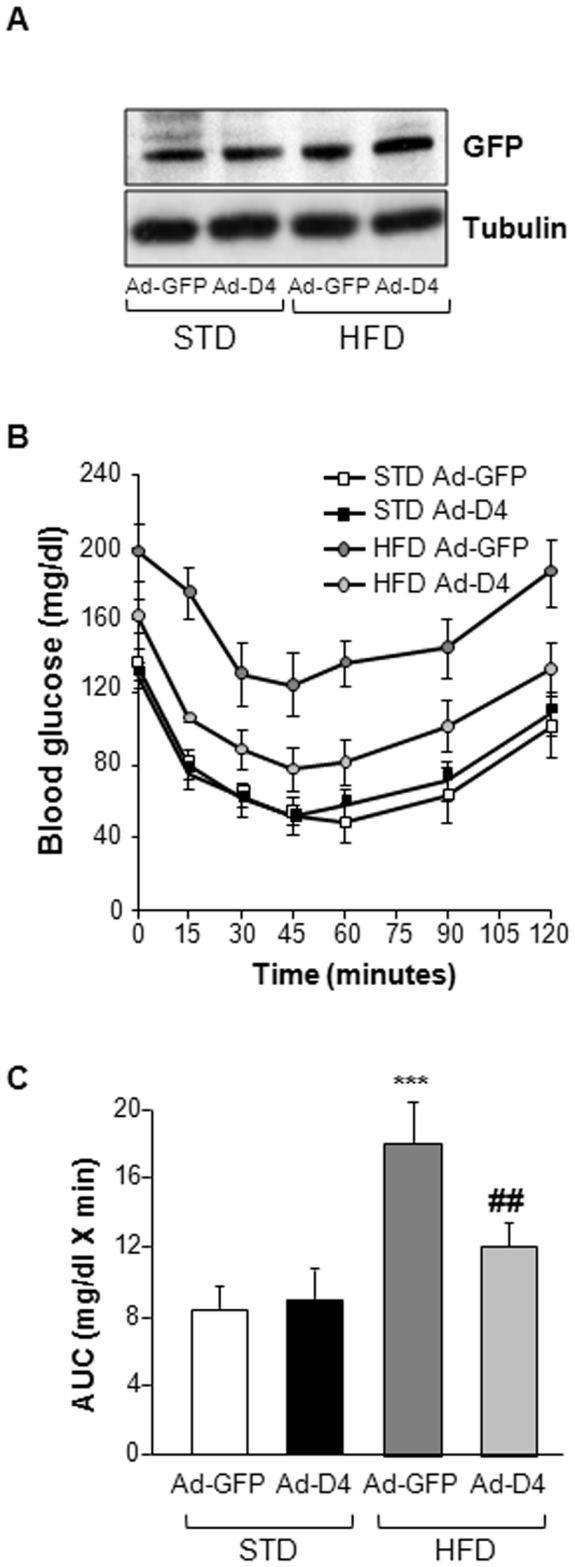
Insulin sensitivity in STD and HFD fed C57BL/6 mice. **A)** Immunoblots of total protein lysates from skeletal muscle homogenates of C57BL/6 mice fed with standard (STD) or high fat (HFD) diets after infection with Ad-GFP or with Ad-D4. The upper blot was probed with anti-GFP antibody, while the bottom blot was probed with anti-Tubulin antibody. **B)** Insulin Tolerance Test (ITT) and (**C**) Area Under the Curve (AUC) glucose in C57BL/6 mice fed with standard (STD, square) or high fat (HFD, circle) diets after infection with Ad-GFP (white and dark gray) or with Ad-D4 (black and light gray). For each experiment, values are expressed as means ± SEM of determinations in at least eight mice per group. ***p<0.001 vs Ad-GFP treated STD fed C57BL/6 mice. ##p<0.01, vs Ad-GFP treated HFD fed C57BL/6 mice.

Recent observation by Ungaro P. et al demonstrated that *Ped/pea-15* mRNA expression is increased by 2-fold in the tibialis skeletal muscle tissue of 6 month old diet-induced obese C57BL/6 mice [14]. Therefore, we investigated if Ad-D4 may improve insulin sensitivity also in this model. To this aim, Ad-D4 was injected in two groups of C57BL/6 mice which were fed either a standard isocaloric diet (STD) or an highly palatable high fat diet (HFD). HFD-fed mice reached a 40% increase of the body weight at the end of the 11 weeks of diet regimen when compared to STD-fed mice and their fasting glycaemia was significantly increased as well (Table 2). D4 levels, measured indirectly by GFP detection, were comparable in tibialis skeletal muscle extracts from C57Bl/6 mice fed with either STD or HFD (Figure 6a). ITT studies revealed that in the HFD-fed mice treated with Ad-GFP glucose levels remained higher during the entire length of the tolerance test, indicating the presence of insulin resistance in these mice (Figure 6b, c). At variance, Ad-D4 treatment strongly improvedinsulin sensitivity in the obese C57Bl/6 mice, as shown by a significant decreased of their blood glucose levels within the 120 min of the test (Figure 6b, c). On the other hand, Ad-D4 treatment did not affect insulin sensitivity of lean STD-fed mice (Figure 6b, c).

**Table 2:** Metabolic characteristics and food intake of HFD and STD fed C57Bl/6 mice.

		*Food Intake*	*Weight*	*Fasting Glucose*
		*(g/day)*	*(g)*	*(mg/dl)*
***STD***	*Baseline*	3.35±0.20	18.7±1.9	73±8
	*w11*	4.29±1.10	27.9±4.4	82±16
	*w11 Ad-GFP*	n.a.	28.3±2.7	84.3±9.2
	*w11 Ad-D4*	n.a.	28.5±1.7	82.9±13.1
***HFD***	*Baseline*	2.97±0.61	18.3±2.2	68±10
	*w11*	4.00±0.90	40.4±5.2^b^	120±15^a^
	*w11 Ad-GFP*	n.a.	41.2±1.9^c^	117±22^c^
	*w11 Ad-D4*	n.a.	41.6±2.3^d^	102±13^e^

Mice were analysed as described under Materials and Methods. Data are the means ± SEM of determinations in 12 STD and 12 HFD fed C57Bl/6 mice on the following conditions: baseline, upon 11 week of diet (w11), upon w11 treated with Ad-GFP and upon w11 treated with Ad-D4. HFD w11 vs. STD w11 mice;

a *p*<0.01 and

b *p*<0.001; HFD w11 Ad-GFP vs. STD w11 mice;

c *p*<0.001; HFD w11 Ad-D4 vs. STD w11 mice;

d *p*<0.001; HFD w11 Ad-D4 vs. HFD w11 Ad-GFP mice;

e *p*<0.001; n.a., not available.

## Discussion

Type 2 Diabetes imposes a major burden on public health at the global level [2]. The pathogenetic heterogeneity of this disorder has long being challenging the development of effective treatment strategies as well as the identification of innovative targets [3], [15].

We have previously identified *PED/PED-15* as a gene that contributes to Type 2 Diabetes susceptibility in humans [4], [5]. In different populations of Type 2 diabetics as well as in the first degree relatives of these individuals [4], [5], PED/PEA-15 was found to be commonly over-expressed in skeletal muscle as well as in white adipose tissues and in peripheral blood leukocytes. Furthermore, PED/PEA-15 protein levels negatively correlate to insulin sensitivity in offsprings of Type 2 diabetics [5]. Studies in cellular and animal models over-expressing PED/PEA-15 have shown that increased PED/PEA-15 expression impairs both insulin action and secretion [4], [6], [7], [13]. While the molecular bases of PED/PEA-15 overexpression have not yet been completely clarified, PED/PEA-15 interaction with PLD1 was identified as a major mechanism responsible for reduced glucose tolerance in animals and humans expressing high PED/PEA-15 levels [8]. Accordingly, we have previously shown that dissociation of PLD1 from PED/PEA-15 ameliorates insulin sensitivity in cultured muscle cells stably overexpressing PED/PEA-15 by reducing basal PKCalpha activity and restoring PKCzeta dependent insulin-stimulated glucose disposal [9]. PCKalpha and PKCzeta are members of the PKC superfamily and play a different role in the regulation of insulin signal transduction. Indeed, activation of the atypical PKC isoforms zeta by insulin is required for the regulation of glucose uptake in adipocytes and in skeletal muscle cells [16], [17], whilst the classical PKCalpha isoform acts both *in vitro* and *vivo* as an endogenous negative feedback inhibitor of insulin signaling by modulating IRS1, PI3K and PKCzeta and lambda as well [18]. In previous work, we showed that blocking PKCalpha either by a specific antisense or by chemical inhibitors as well as the pharmacological inhibition of PLD1 permits recovery of the glucose uptake into muscle and fat cells over-expressing PED/PEA-15 [7], [9]. The effects of PED/PEA-15 on insulin secretion are also reverted by PKCzeta overexpression [13]. However, overexpression of PKCzeta or inhibition of PLD1 or PKCalpha expression/activity are unlikely to represent feasible pharmacological strategies to improve insulin sensitivity and/or secretion due to the pleiotropic roles of these proteins in the regulation of key events in cellular physiology. Furthermore, the existing non–isoform-specific PKC inhibitors interact with other ATP binding kinases and therefore display toxic and severe side effects *in vivo*. [19]–[21]. Alternatively, the inhibition of the interaction between PED/PEA-15 and PLD1 and the development of strategies to convey molecules within the cell that achieve this inhibition may represent an innovative and highly selective approach to improve both insulin sensitivity and beta cell function *in vivo*.

In the present work, we investigated the effect of the PLD1 D4 peptide *in vivo*, using a recombinant adenoviral vector to transduce D4 cDNA (Ad-D4). Adenoviral vectors are very well characterized under the pharmacological, toxicological and pharmacokinetic profile. Many studies in different mouse models demonstrated that they are able to revert phatological phenotype of metabolic disorders like PKU [22] and FH [23]. We have shown that Ad-D4 delivery in mice was well tolerated and led to a widespread expression of the D4 peptide in several tissues, such as skeletal muscle, liver and pancreas. Importantly, we found that the D4 expression restored whole body glucose homeostasis and normalized both fasting and random fed glucose levels as well as fasting insulin and FFA concentration in the Tg_Ped/pea-15 _mice. An increase in fasting serum triglycerol levels and a slight decrease in body weight (data not shown) were observed one week after the adenoviral delivery in both the Ad-D4 and the Ad-GFP treated transgenic mice and interpreted as side effects caused by recombinant adenovirus infection. We also show that D4 expression significantly improved both insulin sensitivity and pancreatic beta-cells insulin secretion in the Tg_Ped/pea-15 _mice, indicating that D4 improvement of glucose tolerance involves actions on insulin target tissues and on pancreatic beta-cells. D4 administration *in vivo* is able to restore basal insulin levels and to improve insulin secretion upon glucose loading. In particular, we observed in Ad-D4 treated Tg_Ped/pea-15 _mice that the secretion of the hormone was slightly increased 3 minutes after glucose loading compared to control mice. Interestingly, this secretory profile is associated with a reduction of the blood glucose levels observed upon 30 minutes from glucose injection during glucose tolerance test, in the Ad-D4 treated Tg_Ped/pea-15 _mice compared to control mice (Figure 2a). Lower glucose levels may be thus responsible for a reduced requirement of hormone release and may explain the difference in the second-phase response in the Ad-D4 treated Tg_Ped/pea-15 _mice. Finally, we confirmed that the over-expression of the D4 peptide in these mice displaced the interaction of PED/PEA-15 with PLD1. After Ad-D4 treatment, the total protein levels of PLD1 in the skeletal muscles from Tg_Ped/pea-15 _mice were similar to those measured in control mice. This, in turns, reduced basal PKCalpha activation, restoring the insulin-stimulated PKCzeta activation and ameliorating glucose disposal in tibialis skeletal muscle. Other molecules implicated in the transduction of insulin signal, such as PI 3-kinase and Akt, apparently, were not affected by the D4 expression (data not shown). These data indicate that, *in vivo*, forced D4 expression impairs PED/PEA-15 binding to PLD1 and may represent a novel strategy to improve insulin sensitivity in skeletal muscle. Together with the positive effect of D4 expression on glucose-induced insulin secretion, this may contribute to the amelioration of whole body glucose homeostasis. Recently, Doti N. et al have shown that the use of a restricted D4 domain, named D4alpha, or the use of short PLD1 peptides that could mimic PED/PEA-15 binding interface can be used as antagonists to prevent the association of PLD1 from PED/PEA-15 [24]. This study provided thus new short, selective and efficient PED/PEA-15-PLD1 antagonists alternative to the D4 that could be transduced to the cells and tissues by adenoviral gene delivery approach. The possibility to vehiculate these smaller compounds and to evaluate their efficacy *in vivo* is currently under investigation in our laboratory.

Next, we have investigated whether over-expression of the D4 peptide is also effective in improving insulin sensitivity in a mouse model of obesity and diabetes. Indeed, recent work from our group has shown over-expression of *Ped/pea-15* mRNA in the tibialis skeletal muscle of C57Bl/6 mice in which obesity is induced by high fat (HFD) feeding [14]. We now report that the D4 treatment improves insulin sensitivity in HFD induced obese C57BL/6 mice, with no effect on the body weight of animals. These findings suggest that i., the efficacy of D4 treatment is not limited to the Tg_Ped/pea-15_ mouse model of insulin resistance; and i.i., *Ped/pea-15* over-expression may play a role in the onset and/or maintenance of insulin resistance during diet-induced obesity.

Whether the efficacy of the D4 treatment in the obese mouse model is exclusively due to PED/PEA-15 increased expression occurring as a consequence of the HFD or is caused also by a different modulation of PLD1 activity still needs to be clarified. PLD1 is a pleiotropic enzyme implicated in several cellular pathways, including signal transduction, membrane trafficking, and the regulation of proliferation and survival [25], and, in addition to PED/PEA-15, many members of the ADP-ribosylation factor (ARF) [26], [27], PKCalpha [28] and small G protein RhoA [29], [30] were identified as PLD interactors and/or activators. Thus, the possibility that the D4 peptide modulates *in vivo* PLD1 activity also by PED/PEA-15-independent mechanisms cannot be excluded.

In conclusion, our findings have established the *in vivo* efficacy of a gene delivery treatment aiming at improving insulin sensitivity by interfering with the interaction between PED/PEA-15 and PLD1. Our results also suggest that this strategy is effective in a mouse model of obesity-induced insulin resistance, and support further efforts aimed at generating novel molecules that interfere with PED/PEA-15 and/or PLD1 functions mimicking D4 peptide action.

## Supporting Information

Figure S1 **Expression of D4 peptide and 2-DG uptake in L6Wt and L6PED/PEA-15 cells.** A) GFP expression was determined in lysates from L6Wt and L6PED/PEA-15 cells after 48h from transduction with Ad-D4 clones 1, 2, 3 and 4. GFP expression was compared with L6PED/PEA-15 transfected with Ad-GFP. Not transfected (NT) cells were used as control. B) Insulin-induced glucose uptake into L6Wt and L6PED/PEA-15 cells not transfected (NT) or transfected with Ad-GFP or Ad-D4 vector. Values for each cell type represent the fold induction upon insulin stimulation over their own basal and are expressed as means ± SEM of determinations in 3 independent experiments. ***p<0.001 vs L6PED/PEA-15 NT.
(TIF)
