# Expression of Concern: Adenoviral Gene Transfer of PLD1-D4 Enhances Insulin Sensitivity in Mice by Disrupting Phospholipase D1 Interaction with PED/PEA-15

**DOI:** 10.1371/journal.pone.0263951

**Published:** 2022-02-08

**Authors:** The PLOS ONE Editors

After this article [1] was published, concerns were raised about western blots in Figs 3C and 4B.

Specifically:

- When adjusted for colour and exposure, it appears that Fig 3C is a composite of four separate images.
- The bands in lanes one and two in the PKCzeta panel in Fig 4B appear similar to the bands in lanes three and four in the PKCzeta panel and to the bands in lanes three and four in the Tubulin panel with different aspect ratios.

In response to queries about these figures, a corresponding author provided the underlying radiograph and replication data for Fig 3C (S1 File). The underlying data confirmed that image fragments were spliced together in preparing Fig 3C but that the fragments originated from the same autoradiograph. The replication data supported the results shown in the figure.

A corresponding author re-reviewed Fig 4B and stated that there are differences between the lanes. They disagree with the concerns raised about Fig 4B and stated that there are differences between all bands shown. The corresponding author stated that the original underlying data for Fig 4B is not available and provided replication data from the original experiments instead (S2 and S3 Files). Due to the unavailability of the original underlying data for Fig 4B, the concerns are not resolved.

In light of the unresolved issues for Fig 4B, the *PLOS ONE* Editors issue this Expression of Concern.

## Supporting information

S1 File Original and replicate data provided in support of Fig 3C. (PDF)

S2 File Replicate data provided in support of Fig 4B. (JPEG)

S3 File Replicate data provided in support of Fig 4B. (JPG)

## References

[1] Cassese A, Raciti GA, Fiory F, Nigro C, Ulianich L, Castanò I, et al. (2013) Adenoviral Gene Transfer of PLD1-D4 Enhances Insulin Sensitivity in Mice by Disrupting Phospholipase D1 Interaction with PED/PEA-15. PLoS ONE 8(4): e60555. doi: 10.1371/journal.pone.0060555 23585839 PMC3621763

