# Supplementary figures and images for: Adenoviral Gene Transfer of PLD1-D4 Enhances Insulin Sensitivity in Mice by Disrupting Phospholipase D1 Interaction with PED/PEA-15
Source: PLoS One. 2013 Apr 9;8(4):e60555. doi: 10.1371/journal.pone.0060555 (PMC3621763; doi:10.1371/journal.pone.0060555)

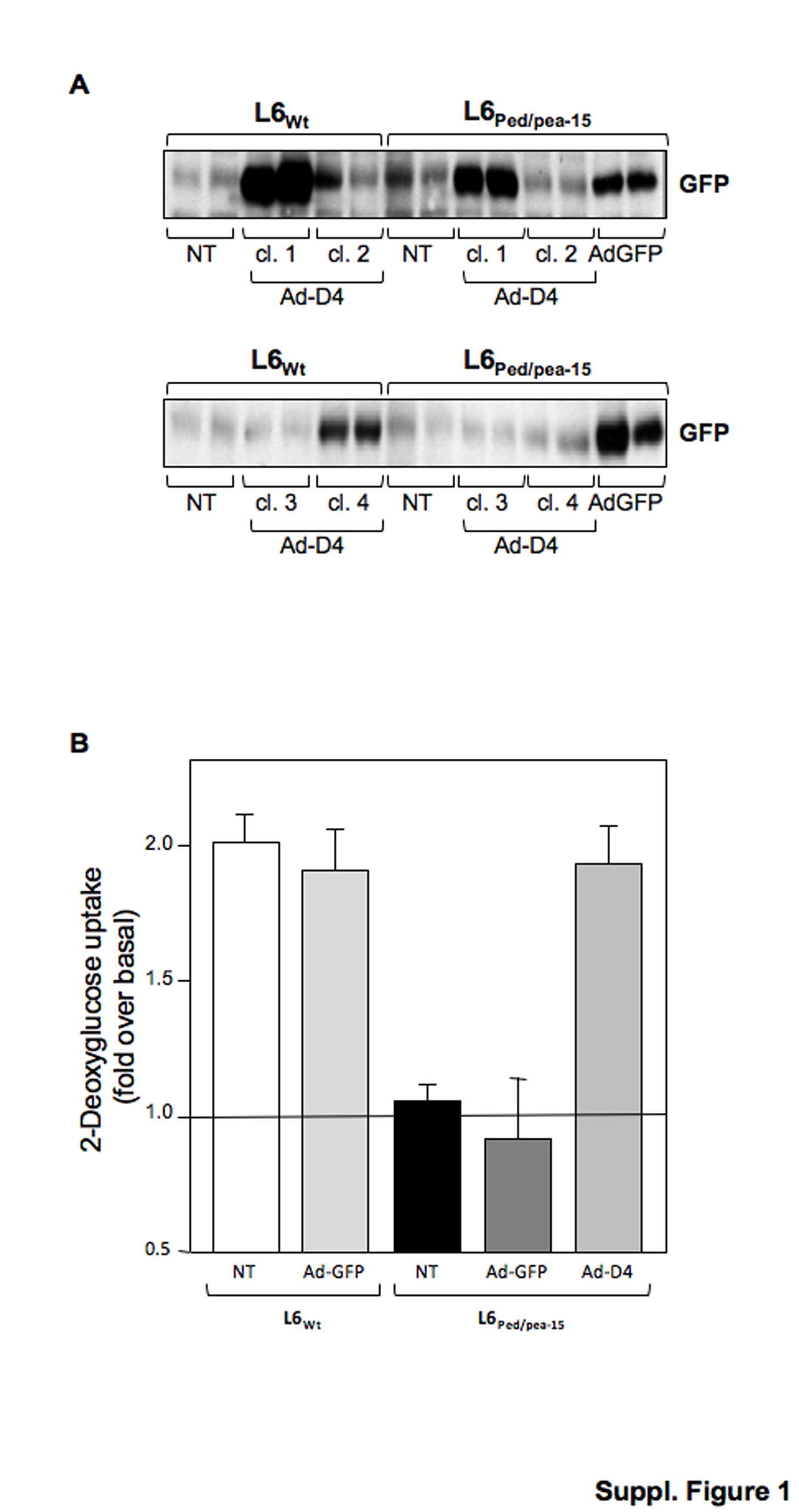

Supplement: Figure S1 — Expression of D4 peptide and 2-DG uptake in L6Wt and L6PED/PEA-15 cells. A) GFP expression was determined in lysates from L6Wt and L6PED/PEA-15 cells after 48h from transduction with Ad-D4 clones 1, 2, 3 and 4. GFP expression was compared with L6PED/PEA-15 transfected with Ad-GFP. Not transfected (NT) cells were used as control. B) Insulin-induced glucose uptake into L6Wt and L6PED/PEA-15 cells not transfected (NT) or transfected with Ad-GFP or Ad-D4 vector. Values for each cell type represent the fold induction upon insulin stimulation over their own basal and are expressed as means ± SEM of determinations in 3 independent experiments. ***p<0.001 vs L6PED/PEA-15 NT. (TIF) [file pone.0060555.s001.tif]
