# Supplementary material for: Expression of Concern: Adenoviral Gene Transfer of PLD1-D4 Enhances Insulin Sensitivity in Mice by Disrupting Phospholipase D1 Interaction with PED/PEA-15
Source: PLoS One. 2022 Feb 8;17(2):e0263951. doi: 10.1371/journal.pone.0263951 (PMC8824318; doi:10.1371/journal.pone.0263951)
Supplement: S1 File — (PDF) [file pone.0263951.s001.pdf]

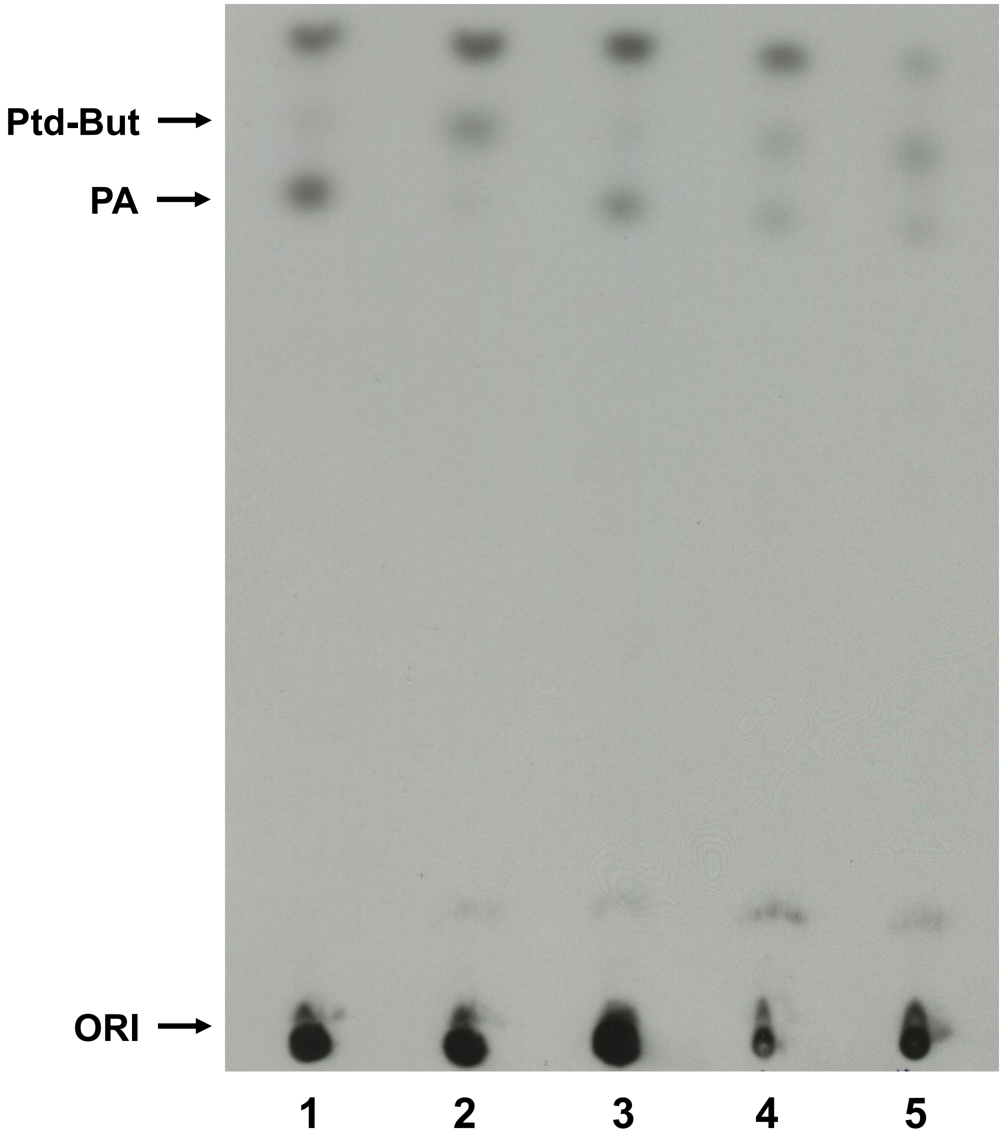

Original experiment shown in Figure 3C

Legend

1: Wt 1 (skeletal muscle)
2: $Tg_{Ped/pea-15}$ 2 (skeletal muscle)
3: L6 skeletal muscle cells (untreated)
4: L6 skeletal muscle cells (insulin - stimulated)
5: L6 skeletal muscle cells (PMA - stimulated)

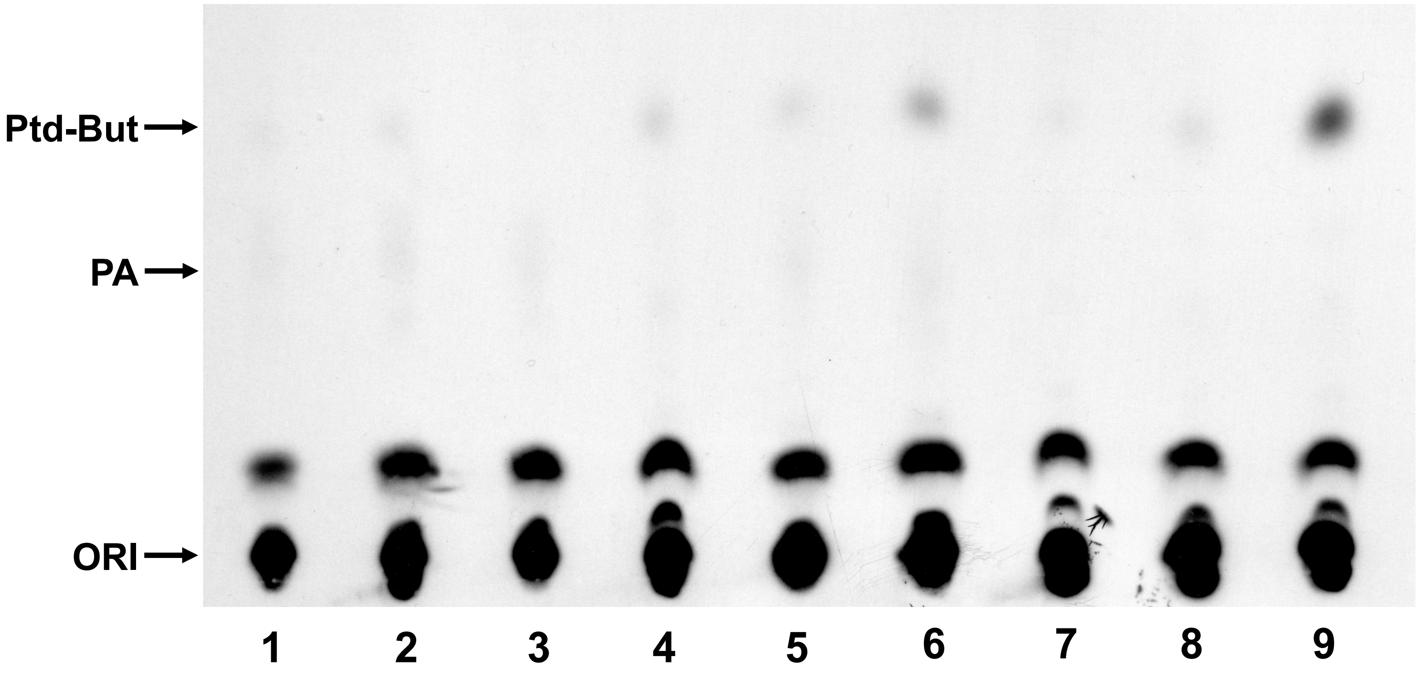

Duplicate #1 of the experiment shown in Figure 3C

Legend

1: L6 skeletal muscle cells (untreated)
2: L6 skeletal muscle cells (insulin – stimulated)
3: L6 skeletal muscle cells (untreated)
4: L6 skeletal muscle cells (insulin – stimulated)
5: Wt 3 (skeletal muscle)
6: Tg$_{\mathit{Ped/pea\text{-}15}}$ 4 (skeletal muscle)
7: Wt 5 (skeletal muscle)
8: Wt 6 (skeletal muscle)
9: Tg$_{\mathit{Ped/pea\text{-}15}}$ 7 (skeletal muscle)

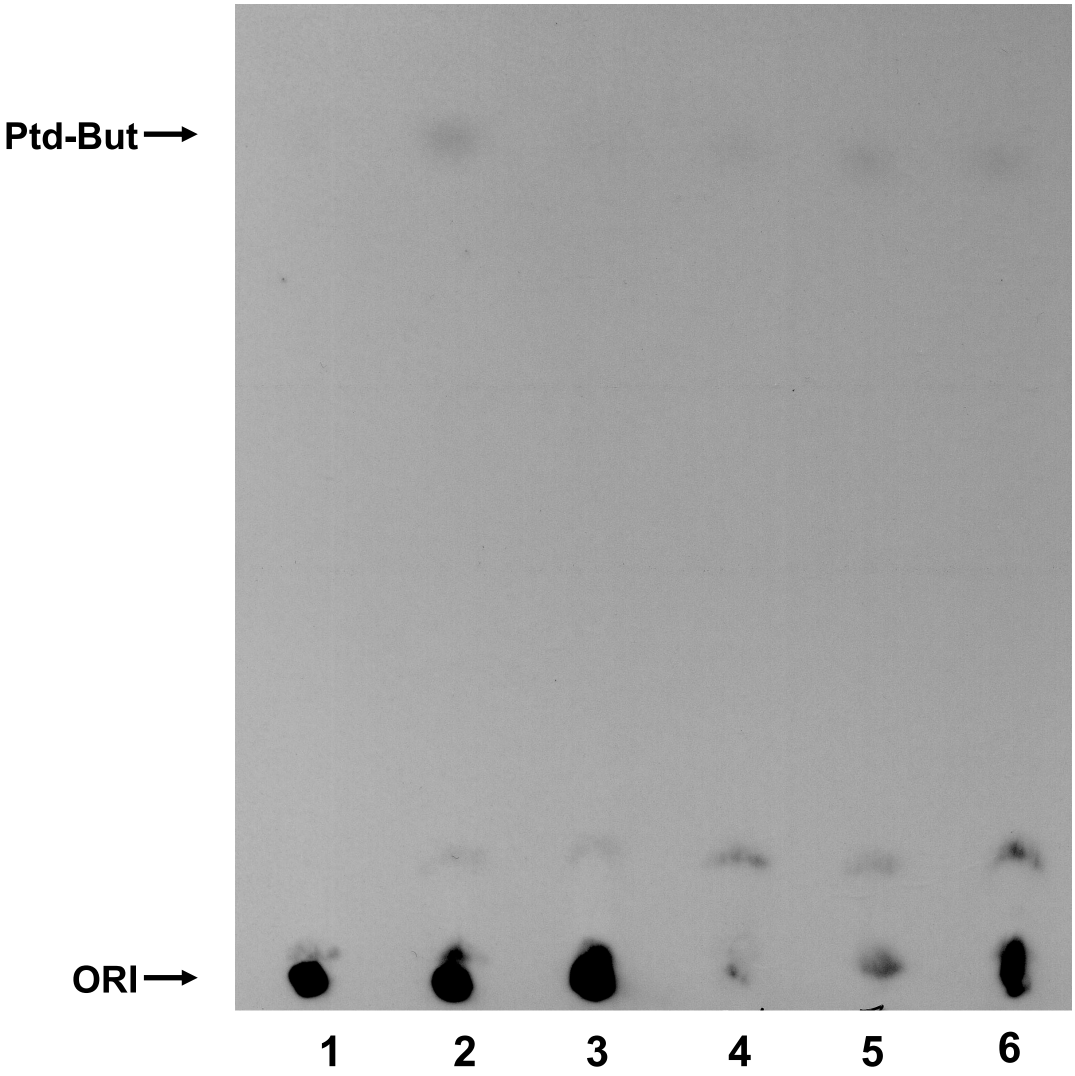

Duplicate #2 of the experiment shown in Figure 3C

Legend

1: L6 skeletal muscle cells (untreated)
2: L6 skeletal muscle cells (insulin – stimulated)
3: Wt 8 (skeletal muscle)
4: Wt 8 (skeletal muscle)
5: Tg$_{Ped/pea-15}$ 9 (skeletal muscle)
6: Tg$_{Ped/pea-15}$ 9 (skeletal muscle)
