# Supplementary figures and images for: Expression of Concern: Adenoviral Gene Transfer of PLD1-D4 Enhances Insulin Sensitivity in Mice by Disrupting Phospholipase D1 Interaction with PED/PEA-15
Source: PLoS One. 2022 Feb 8;17(2):e0263951. doi: 10.1371/journal.pone.0263951 (PMC8824318; doi:10.1371/journal.pone.0263951)

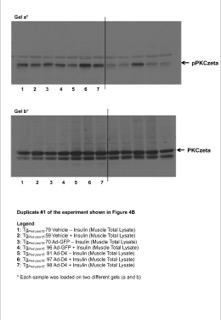

Supplement: S2 File — (JPEG) [file pone.0263951.s002.jpeg]

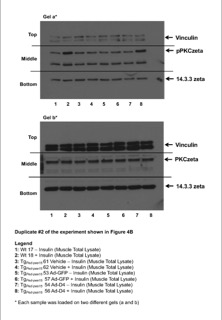

Supplement: S3 File — (JPG) [file pone.0263951.s003.jpg]
